# High Moisture Accelerated Mechanical Behavior Degradation of Phosphor/Silicone Composites Used in White Light-Emitting Diodes

**DOI:** 10.3390/polym11081277

**Published:** 2019-07-31

**Authors:** Jiajie Fan, Zhen Wang, Xunwei Zhang, Zhentao Deng, Xuejun Fan, Guoqi Zhang

**Affiliations:** 1 College of Mechanical and Electrical Engineering, Hohai University, Changzhou 213022, China; 2 State Key Lab of Solid State Lighting, Changzhou Institute of Technology Research for Solid State Lighting, Changzhou 213164, China; 3 Department of Microelectronics, EEMCS Faculty, Delft University of Technology, 2628 Delft, The Netherlands; 4 College of Chemistry and Environmental Engineering, Shenzhen University, Shenzhen 518061, China; 5 Department of Mechanical Engineering, Lamar University, Beaumont, TX 77710, USA

**Keywords:** light-emitting diode, phosphor/silicone composite, curing mechanism, mechanical properties, moisture

## Abstract

In a high-power white light emitting diode (LED) package, the phosphor/silicone composite is typically used for photometric and colorimetric conversions, ultimately producing the white light. However, the phosphor/silicone composite is always exposed under harsh environments with high temperature, high blue light irradiation and high moisture when the LED operates. Therefore, its reliability issue has become one of the critical bottlenecks to improve the lifetime of a high-power white LED package. As the curing process and mechanical behavior of phosphor/silicone composite essentially determine its reliability, this paper firstly uses an in situ viscosity monitoring approach combined with Differential Scanning Calorimetry (DSC) and Fourier Transform Infrared Spectroscopy (FTIR) analysis to explain the curing mechanism of a phosphor/silicone composite by taking the effects of temperature and phosphor mass fraction into consideration. Then, the mechanical properties of phosphor/silicone composites aged under a long-term high moisture condition are evaluated by using the tensile test. Meanwhile, the finite element (FE) simulations, the Mori–Tanaka theoretical estimations and the microstructure analysis are applied to investigate the high moisture induced degradation mechanisms. The results show that: (1) the in situ measured isothermal viscosity curves of both pristine silicone and phosphor/silicone composites follow the Arrhenius empirical model, and high temperature and high phosphor mass fraction can increase the curing rate; (2) the hydrosilylation reaction between silicones determines the curing mechanism of phosphor/silicone composite; (3) the tensile test, FE simulation and Mori–Tanaka theoretical prediction results confirm that the Young’s modulus of phosphor/silicone composite increases by gradually adding phosphors; and (4) the Young’s modulus of phosphor/silicone composite increases after the high moisture ageing test, which can be attributed to the oxidation and cross-linking reaction of silicone and the hydrolysis of phosphor powders.

## 1. Introduction

A high-power white light emitting diode (LED) has been considered as a new generation of light source widely used in our daily life due to their high luminous efficiency, low energy consumption, long life and no pollution. At present, the most popular white LED package is a combination of a blue LED chip with yellow phosphors and its white light luminous mechanism is that the blue light generated by the LED chip mixes with the yellow light excited by phosphors [1,2]. In this phosphor-converted white LED (pc-WLED) package, phosphors and silicone encapsulant are usually combined as a composite to undertake the functions of both blue light down-conversion and chip protection [3]. However, when being used in the harsh environment (e.g., high temperature and high moisture conditions) [4], the phosphor/silicone composites will obviously deteriorate especially in case of the insufficient curing process, which can result in a serious lumen degradation and color shift of a pc-WLED package [5,6].

Many previous studies indicate that the curing process of phosphor/silicone composite can directly affect the light conversion efficiency, thermal dissipation and reliability of pc-WLEDs [7,8,9]. For example, Hou et al. [10] synthesized a siloxane-type epoxy resin by the hydrosilylation reaction, which had pendant epoxide rings on the side chain of the polysiloxane polymer backbone. The curing process was investigated by the Differential Scanning Calorimetry (DSC) analysis, which showed that the addition of a siloxane-type copolymer into epoxy resin could increase the initial curing temperature and peak curing temperature. Khalilullah et al. [11] found that the moisture could cause the irreversible swelling of phosphor/silicone composite due to its insufficient curing process. Wei et al. [12,13] pointed out that it was possible to improve the curing effect by adjusting the curing conditions, such as increasing the temperature, ultraviolet illumination, etc. Wang et al. [14] studied the effects of temperature and ultraviolet illumination on the curing process of the phosphor/silicone composite, and reported its curing kinetic mechanisms. It is found that the curing process followed the Arrhenius model and the ultraviolet light could accelerate the curing reaction. In addition, several researchers have reported the accelerated ageing of polymer based LED packaging materials. Luo et al. [15] compared the degradation mechanisms of phosphor/silicone composites used in pc-WLEDs under both high temperature and high humidity conditions. The results show that the hydrolyzation of phosphors and the oxidation of silicone under a high moisture environment could accelerate the degradation of phosphor/silicone composites. Singh et al. [16] reported the lumen degradation of high-power LEDs aged under high-humidity testing and observed the lumen recovery after a sharp drop of lumen maintenance. The finite element method was used to simulate the moisture absorption–desorption process in a LED package to understand this recovery phenomenon. This phenomenon was explained due to the increasing moisture absorption of silicone resulting in subsequent light scattering. Fan et al. [17] studied the degradation mechanism of LED phosphor powders under a hygrothermal environment, which reveals that the photoluminescence intensity, color purity and light conversion efficiency of phosphors decreased and their surface temperatures increased due to the hydrolysis reaction that occurred.

The high temperature and high humidity conditions always have critical impacts on the mechanical properties of phosphor/silicone composites; these are related to the reliability of pc-WLED packages. Some researchers have touched on this problem, such as Zhang et al. [18], who used the Dynamic Mechanical Analysis (DMA) approach to study the dynamic mechanical properties of silicone resins and found that glass transition temperature was found at 40 °C, and the frequency increased when the glass transition temperature increases. Moreover, factors such as storage modulus, loss factor, and glass transition temperature of phosphor/silicone composites were characterized to improve the reliability of pc-WLEDs. Wang et al. [19] studied the mechanical properties of silicone rubber and presented its storage life prediction model by using the dichotomy and linear regression estimation method. Gac et al. [20] explained the ageing mechanism and mechanical degradation of polychloroprene rubber in the marine environment. In the study, the tensile properties and microhardness of polychloroprene rubber were tested to evaluate its mechanical properties. The results show that the stiffness of sample under accelerated ageing and natural ageing increased and its modulus had a mutation process and the reason for the mutation was concluded as the formation of Si-OH. Chen et al. [21,22] revealed that the mechanical properties of silicone/phosphor composites had a great influence on the lifetime of LED products, as their mechanical properties were highly sensitive to the high temperature and high humidity conditions [23,24,25]. Chen et al. [26,27] studied the effects of different phosphor mass fractions on the mechanical properties of phosphor/silicone composites by using the tensile tests and microscopic numerical simulations. They concluded that the addition of phosphors could shorten the average distance between phosphor particles and intensified the strain localization in the silicone matrix, which consequently results in the delamination between phosphors and silicone. Pan et al. [28] tested the mechanical properties of phosphor/silicone composites with different phosphor mass fractions aged under a high temperature (85 °C) condition. Their results reveal that the Young’s modulus increased with the increasing of phosphor mass fraction in silicone, and the high temperature ageing could stiffen the composite and weaken its ductility.

As reviewed above, some scholars have studied the effects of phosphor mass fraction and temperature on the mechanical properties of phosphor/silicone composites. However, few scholars consider the curing mechanism of phosphor/silicone composite and the relationship to its mechanical behaviors, especially aged under a long-term high moisture condition. Since both the curing process and mechanical properties of phosphor/silicone composite will affect the reliability of the LED package, the purpose of this paper is to fundamentally understand the curing mechanism and the mechanical degradation mechanism of a phosphor/silicone composite under high humidity conditions. The major contributions of this paper are to support the theoretical basics for the LED packaging material selection and process optimization, and also to provide the technical guidance on the reliability assessment for high quality LED light sources.

## 2. Sample Preparation and Experimental Setup

In this section, a curing experiment was designed in advance to prepare the test samples used in this study. Then, a long-term high moisture accelerated ageing test was conducted for the prepared test samples. During the ageing experiment, the mechanical properties of test samples were measured by using the tensile testing.

### 2.1. Sample Preparation

**Figure 1:**
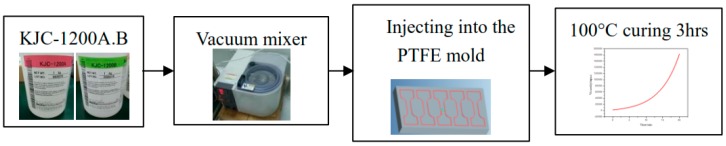
Test sample preparation procedure.

**Figure 2:**
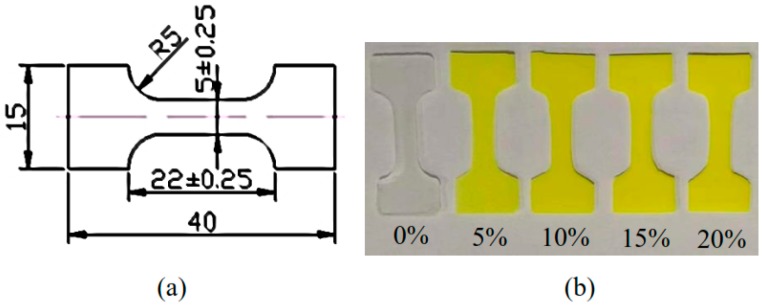
(**a**) geometric dimension of test samples; (**b**) the prepared test samples with different phosphor mass fractions.

The preparation procedure of test sample used in this paper follows the general process recommended in LED CSP packaging. As shown in Figure 1, firstly, the silicones KJC-1200A and KJC-1200B from Shin-Etsu Chemical Co. Ltd. (Tokyo, Japan) were mixed with a 1:1 mass ratio. Next, the silicone mixture and the YAG yellow phosphor powders (Type: YGFA13) with different mass fractions were thoroughly mixed in a vacuum mixer, and the phosphor/silicone mixture was then poured into a polyfluortetraethylene (PTFE) mold. Finally, the phosphor/silicone mixture was cured in a 100 °C oven for 3 h, cooled and taken out. The geometric dimensions of test samples with a thickness of 1 mm are designed according to the ASTM D1708 standard [29] as shown in Figure 2a,b, which presents the test samples with different phosphor mass fractions.

### 2.2. Experimental Setup

**Figure 3:**
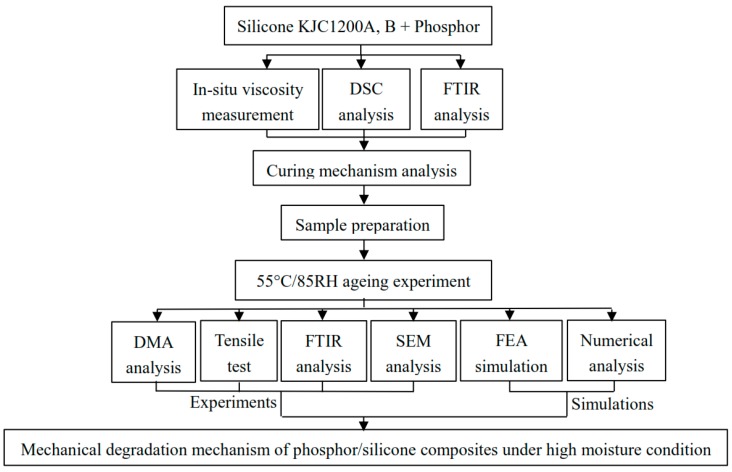
The flowchart of the study plan.

The flowchart of study plan in this paper is shown in Figure 3. Two major parts include the analysis of both curing mechanism and long-term high moisture accelerated mechanical degradation mechanism. In detail, the in situ viscosity monitoring, DSC and FTIR test are used to analyze the curing mechanism of silicone, while the tensile strength, DMA, FTIR, and Scanning Electron Microscope/ Energy Dispersive X-Ray Spectroscopy (SEM/EDS) tests are used to analyze the degradation mechanism of prepared test samples. Furthermore, both FEA simulation and theorical calculation are used in this study to explain the mechanical properties of phosphor/silicone composites.

**Figure 4:**
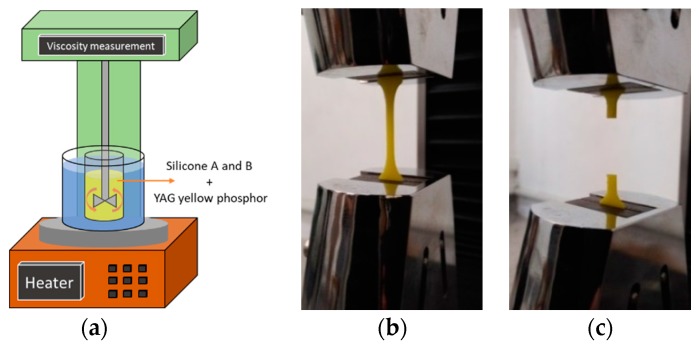
(**a**) the curing experimental setup; and tensile testing setup; (**b**) before testing; (**c**) after testing.

The curing experiment of phosphor/silicone composites comprehensively considers the influence of temperature (65 °C, 80 °C, 95 °C) and phosphor concentration (0%, 5%, 10%) in the curing process. The curing experimental setup is shown in Figure 4a. The viscosities of composites were in situ measured by the viscosity measurement instrument (Model: NDJ-8S, accuracy: ±1%, the rotating speed is set as 30 r.p.m.) during the curing process. The high moisture ageing test condition was designed by considering the similar high temperature and high humidity condition as the summer weather in south China (55 °C/85RH). The prepared test samples were placed in the constant temperature and humidity chamber with 1008 h ageing time. Five test samples per group were picked out for the tensile test, DMA and FTIR characterizations at every 168 h. The tensile test setup with an Electromechanical Universal Testing Machine from the MTS system Co. Ltd. (Shenzhen, China) (Model: CMT4204, accuracy: level 0.5) is shown in Figure 4b,c.

## 3. Results and Discussion

In this section, the test and simulation results of test samples under curing experiment and ageing experiment are completely analyzed and discussed.

### 3.1. Curing Mechanism Analysis

In this study, the curing mechanism of phosphor/silicone composites was analyzed by using the in situ viscosity, DSC and FTIR measurements.

#### 3.1.1. In Situ Viscosity Measurement Results and Discussion

**Figure 5:**
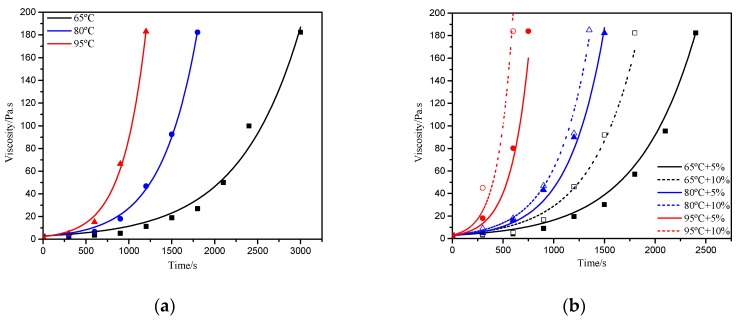
In situ viscosity measurement data of (**a**) pure silicone and (**b**) phosphor/silicone composites and their fitting results by the Arrhenius model.

The effects of temperature and phosphor mass fraction were considered into the curing process of phosphor/silicone composites, which can be described by an isothermal rheological model shown in Equation (1). The in situ measured viscosity data are fitted with Equation (1) and shown in Figure 5:

(1) $$\mathrm{In}\eta =\mathrm{In}\eta_{\infty }+\frac{E_{\eta }}{RT}+\;tK_{\infty }\exp\left(-\frac{E_{K}}{RT}\right),$$

where *η*_∞_ is the viscosity of silicone when *T* = ∞ and *E_η_* is the viscous flow activation energy, *K*_∞_ is the kinetic parameter corresponding to *η*_∞_, and *E_K_* is the kinetic parameter corresponding to *E*_η_, the constant *R* = 8.314 J/mol·K.

As can be seen from Figure 5, when the temperature and the mass fraction of phosphor increase, the increment velocity of viscosity increases, which means that the solidification reaction rate increases. The temperature dependent Arrhenius model described in Equation (1) fits the experimental data well, which indicates that the curing processes of both pure silicone and phosphor/silicone composites can be modelled by the Arrhenius equation.

#### 3.1.2. DSC and FTIR Results and Discussion

**Figure 6:**
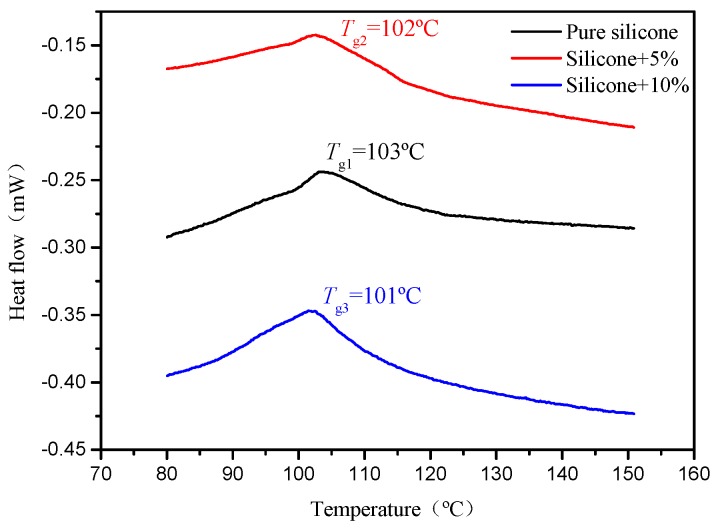
DSC measurement results of pure silicone and phosphor/silicone composites.

The glass transition temperatures, *T*_g_, of pure silicone and phosphor/silicone composites with mass fractions of 5% and 10% were tested by using the DSC, respectively. As shown in Figure 6, the *T*_g_ of both pure silicone and phosphor/silicone composites are extracted around 103 °C. This can be explained by the fact that, as there is large difference of *T*_g_ between phosphor and silicone and the addition of phosphor will not break the molecule chain of silicone, the *T*_g1_ of silicone plays a dominant role in the *T*_g2_ and *T*_g3_ of prepared 5% and 10% phosphor/silicone composites. Thus, both pure silicone and phosphor/silicone composites can be cured under the same condition to prepare the test samples.

**Figure 7:**
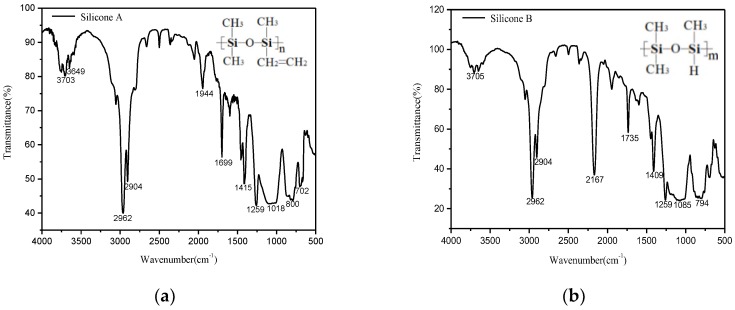
The FTIR spectrum of silicones and their molecular formula (**a**) silicone A; (**b**) silicone B.

Next, in order to figure out the curing mechanism of silicone, the FTIR tests for silicones A and B before and after curing were used to determine the changes of their molecular structures. By analyzing the FTIR test results of silicone A and silicone B shown in Table 1, their molecular formula can be determined as shown in Figure 7.

**Figure 8:**
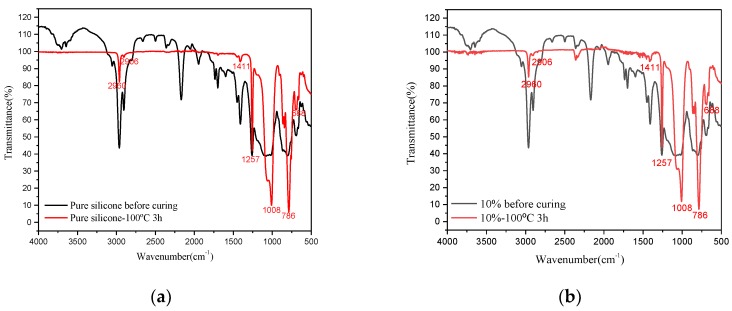
FTIR results of (**a**) pure silicone and (**b**) phosphor/silicone composites before and after curing at 100 °C for 3 h.

**Scheme 1:**
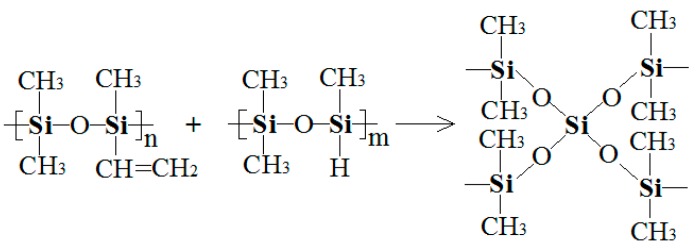
Curing reaction mechanism of silicone A and B.

Figure 8 compares the FTIR characteristic absorption peaks of pure silicone and phosphor/silicone composites before and after curing at 100 °C for 3 h. It shows that the silicon hydrogen bonds between 2161 cm^−1^ and 1680–1620 cm^−1^ and the vinyl bonds between 1008 cm^−1^ and 786 cm^−1^ disappeared after curing. However, there is a strong characteristic absorption peak of silicon siloxane bond, which indicates that the silicone mixture has been substantially solidified after curing. According to the above experimental results, it is speculated that a hydrosilylation reaction occurs in the curing process of silicone A and B, and its curing reaction mechanism can be referred to Scheme 1.

### 3.2. Moisture-Accelerated Degradation Mechanism Analysis

The cured test samples, including pure silicone and phosphor/silicone composites with mass fraction of phosphor as 10%, were conducted a 1008 h ageing at the condition of 55 °C/85RH. In this section, their mechanical properties were evaluated according to the FEA simulations, theorical calculations and long-term tensile testing. The moisture accelerated degradation mechanism was analyzed by the FTIR, DMA and SEM/EDS measurements.

#### 3.2.1. Transient Mechanical Property Prediction with FEA Simulations

**Figure 9:**
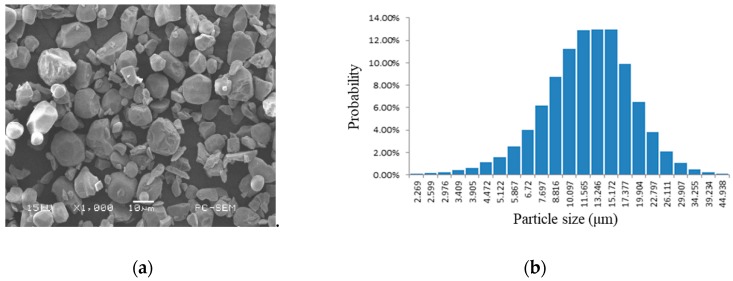
(**a**) SEM image of phosphor particles and (**b**) its diameter distribution.

In order to study the effect of microstructure on the macroscopic mechanical properties of phosphor/silicone composites, a random multi-sphere model was used in this study to simulate the transient mechanical properties of composites. Figure 9a is the YAG yellow phosphor particle photographed by an scanning electron microscope, and it is observed that the particle diameters are not completely uniform and conform to a statistical distribution as shown in Figure 9b. According to the statistical analysis, it is known that the particle diameter distribution of phosphor particles can be expressed as a lognormal function (Equation (2)). As shown in Figure 9b, the standard deviation and mean of particle diameter can be obtained by fitting Equation (2) [26]:

(2) $$f(r)=\frac{1}{rs\sqrt{2\pi }}\exp^{-\frac{{\left(In(r)-\mu \right)}^{2}}{2s^{2}}},$$

where *r* represents a random variable of the sphere radius, and *μ* and *s* represent the mean and standard deviation, respectively. As calculated, the standard deviation of particle diameters is 4.57 μm and the mean of particle diameters is 11.32 μm.

**Figure 10:**
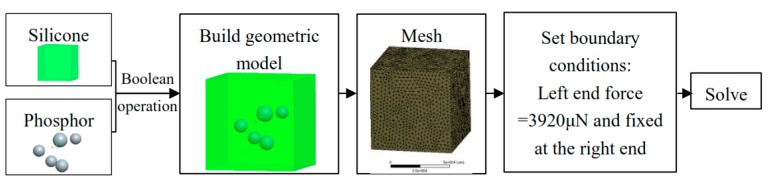
FEA modeling and simulation process.

**Figure 11:**
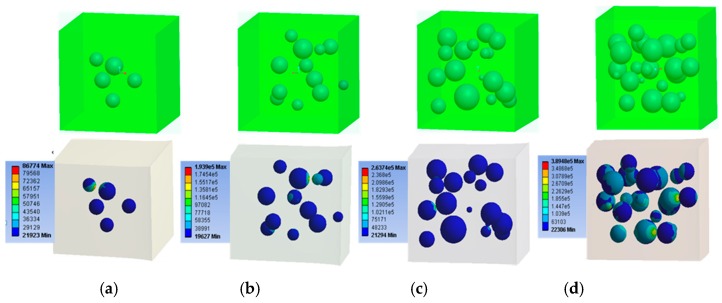
Volume element models and transient mechanical simulation results of phosphor/silicone composites (**a**) 5%; (**b**) 10%; (**c**) 15%; (**d**) 20%.

In the FEA modeling and simulation process as shown in Figure 10, the representative volume element model has a size of 70 μm × 70 μm × 70 μm in which the randomly distributed spherical particles represent phosphor powders. The cube model represents the silicone matrix and it adopts the superelastic neo-Hookean constitutive relation. The component models of phosphor particles and silicone are assembled by using the Boolean operations. The phosphor particles are randomly distributed in a unit volume element based on a certain statistical law and it must ensure that there is no overlap that occurs between the particles. The simulation parameters of components at the initial state are shown in Table 2 and the specific simulation process is described in Figure 10. Figure 11 presents the volume element models and transient mechanical simulation results of phosphor/silicone composites with mass fractions of 5%, 10%, 15% and 20%, respectively.

#### 3.2.2. Transient Mechanical Property Prediction with the Mori–Tanaka Method

When the material properties of particles and matrix and the volume fraction of particles in the matrix are known, the equivalent mechanical properties of composite can be theoretically predicted in the elastic range. The commonly used analytical estimation theories include the Mori–Tanaka, the Self-Consistent and the differential methods [30]. As described in the Mori–Tanaka method [31], for composite feature units, when the inclusions are inter-influenced, the multi-inclusion problem can be converted into a single inclusion problem, and the far field strain value in the single inclusion problem could be set to the average strain of the composite matrix portion. Thus, the Mori–Tanaka model can be used to estimate an equivalent elastic modulus of a composite being expressed as follows:

(3) $$L=L_{0}{\left(I+CA\right)}^{-1},$$

(4) $$A=\{L_{0}+(L_{2}-L_{0})[CI+(1-C)S]{\}}^{-1}(L_{0}-L_{2}),$$

where *L*_2_ is an elastic constant of the inclusion phase; *I* is Fourth-order unit tensor; and *S* is the fourth-order Eshelby tensor.

For spherical particle composites, Equation (3) can be simplified to:

(5) $$K=K_{m}\left[1+\frac{C(K_{2}-K_{m})}{K_{m}+\alpha (1-C)(K_{2}-K_{m})}\right],$$

(6) $$\mu =\mu_{m}\left[1+\frac{C(\mu_{2}-\mu_{m})}{\mu_{m}+\beta (1-C)(\mu_{2}-\mu_{m})}\right],$$

(7) $$\alpha =\frac{1}{3}(\frac{1+\gamma_{0}}{1-\gamma_{0}}),$$

(8) $$\beta =\frac{2}{15}(\frac{4-5\gamma_{0}}{1-\gamma_{0}}),$$

(9) $$E=\frac{9K\mu }{3K+\mu },$$

where *K* and *μ* are the bulk modulus and shear modulus of the composite; *K_m_* and *μ_m_* are the bulk modulus and shear modulus of the matrix material; *K*_2_ and *μ*_2_ are the bulk modulus and shear modulus of the inclusions; *C* is volume content of inclusion particles; *γ*_0_ is Poisson’s ratio of matrix material; and *E* is effective Young’s Modulus of composites.

**Figure 12:**
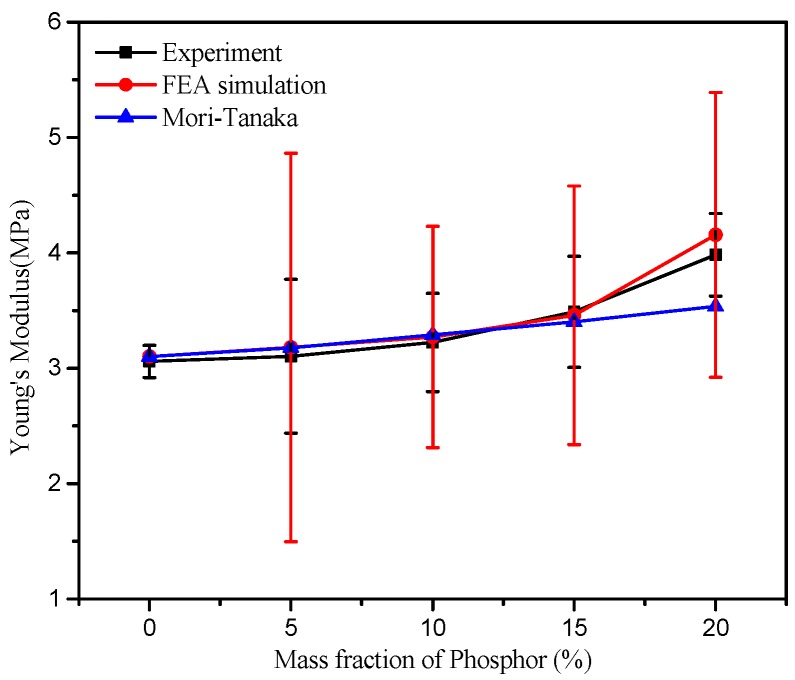
Comparison of transient Young’s modulus results from FEA simulations, Mori–Tanaka calculations and tensile tests.

By comparison, the transient Young’s modulus of phosphor/silicone composites with mass fraction of 5%, 10%, 15% and 20% were estimated by using both the FEA simulation and the Mori–Tanaka method. The estimated results were compared to the actual tensile testing results. Firstly, the averaged Young’s modulus of ten randomly generated models from each mass fraction group were simulated with the FEA method described in Section 3.2.1. As indicated in Figure 12, it can be found that, as the phosphor mass fraction increases, the averaged Young’s modulus increases gradually, which is consistent with the experimental results. In addition, the Young’s modulus obtained from experiment is relatively lower than that from simulation, which may be due to the fact that the phosphor has a certain degree of precipitation during the composite’s preparation [27]. Secondly, when it compares to the theoretical calculation based on the Mori–Tanaka method, it shows that, when the phosphor mass fraction is small, both the FEA simulation results and Mori–Tanaka calculations are close to experimental results. However, when the phosphor mass fraction increases, the gaps between the FEA simulation results and the Mori–Tanaka calculations become larger. This indicates that the Mori–Tanaka method is suitable to predict the mechanical properties of phosphor/silicone composites with low phosphor contents. This is because, when the phosphor content is increased, the uneven distribution of phosphor powders in the silicone matrix results in the gap between theoretical calculations and experiments.

#### 3.2.3. Long-Term Tensile Test Result Analysis

**Figure 13:**
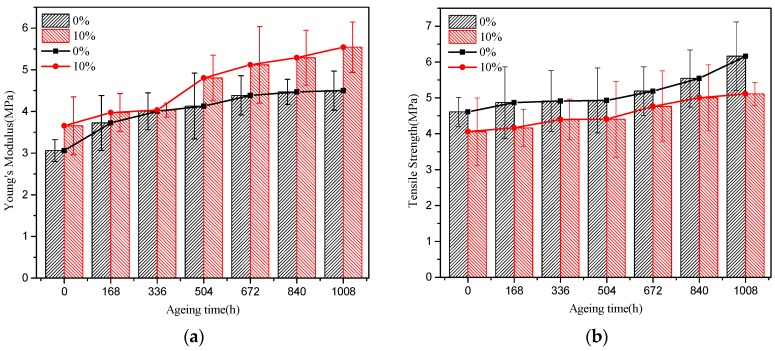
The tensile test results (**a**) Young’s modulus; (**b**) tensile strength.

The ageing test for samples with 0% and 10% phosphor mass fraction, through periodic tensile testing, the Young’s modulus and tensile strength of test samples aged under a long-term high moisture condition were obtained and shown in Figure 13. Figure 13a shows that, during the ageing process, the Young’s modulus of both pure silicone and phosphor/silicone composites increased, which means they became stiffer after ageing. Moreover, the Young’s modulus increment rate of the phosphor/silicone composite is larger than that of pure silicone. It is speculated that, when the moisture permeated into the silicone, the hydrolysis reaction of phosphor powders accelerated the stiffness of composite. The tensile strength is defined as the maximum stress divided by the cross-sectional area. It is observed in Figure 13b that the tensile strengths of both pure silicone and phosphor/silicone composites were gradually increased during the ageing process. However, the tensile strength increment of pure silicone samples is higher than that of phosphor/silicone composites, especially at the end of ageing. This can be explained as the reason that the moisture induced hydrolysis of phosphor powders increased their surface roughness, resulting in the uneven contact between phosphor particles and the silicone. This might produce gaps between them and slow down the tensile strength increment rate of composite.

**Figure 14:**
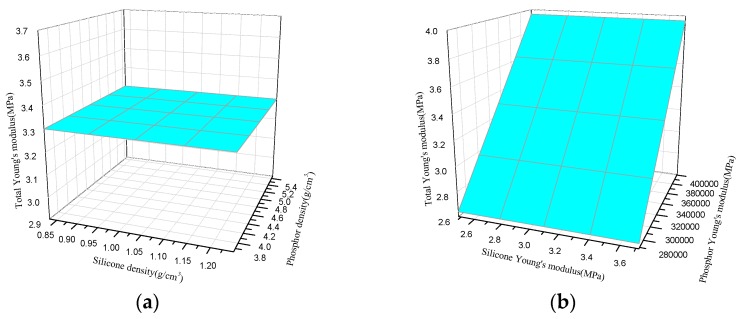

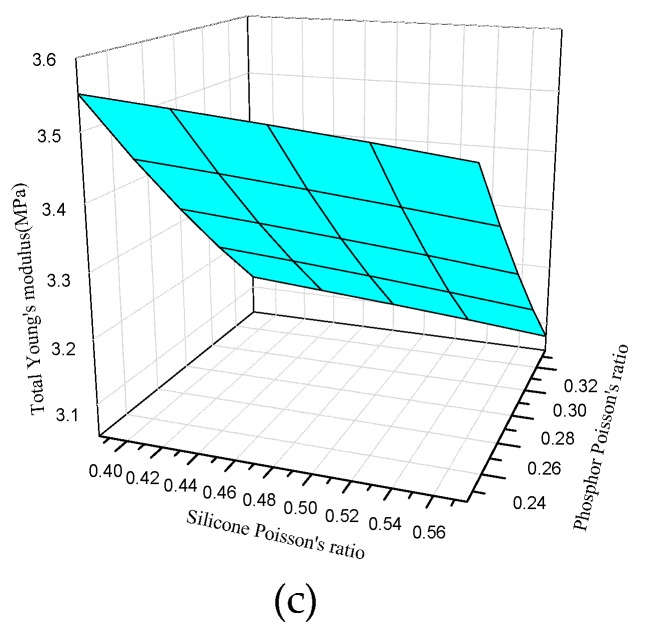
A sensitivity study on the Young’s modulus of phosphor/silicone composites (**a**) density; (**b**) Young’s modulus; (**c**) Poisson’s ratio.

Due to the density, Young’s modulus and Poisson’s ratio of phosphor powders not being able to be accurately known during the ageing process, a sensitivity study was conducted in this section to quantitively evaluate the impact of component’s parameters on the Young’s Modulus of phosphor/silicone composites by using the FE simulation method. The simulation results are shown in Figure 14. It can be summarized from Figure 14a that the densities of both silicone and phosphor have less impact on the total Young’s modulus of composite. Figure 14b,c reveal that the Young’s modulus and the Poisson’s ratio of phosphor powders can determine that of composites.

#### 3.2.4. DMA and FTIR Result Analysis

**Figure 15:**
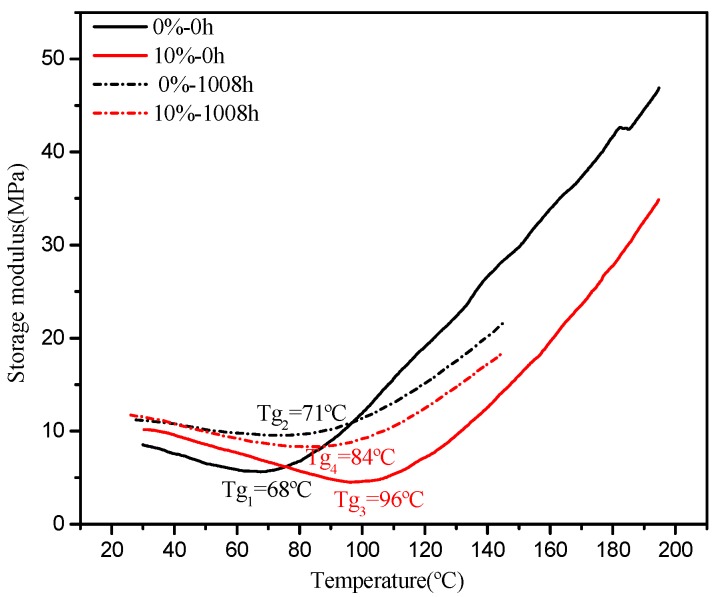
DMA test results before and after 1008 h high moisture ageing test.

Moreover, to explain the degradation mechanism of phosphor/silicone composites aged under high moisture conditions, the DMA test was firstly implemented on the test samples before and after 1008 h of ageing. The results are shown in Figure 15, which presents that the storage modulus of both pure silicone and phosphor/silicone composites increased after high moisture ageing, when the operation temperature was under their glass transition temperatures, which means that the elastic deformation storage abilities of all test samples after high moisture ageing were enhanced. This result keeps consistent with the increment of Young’s modulus from the tensile test. In addition, after 1008 h ageing, the glass transition temperature of pure silicone increased from 68 to 71 °C, but that of phosphor/silicone composite decreased from 96 to 84 °C. This may be attributed to the hydrolysis of phosphor powders during the ageing, increasing the stiffness of phosphor/silicone composite.

**Figure 16:**
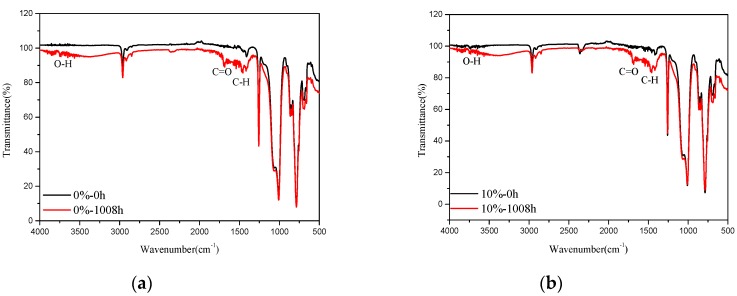
FTIR results of (**a**) silicone and (**b**) 10% phosphor/silicone composite before and after 1008 h high moisture ageing test.

**Scheme 2:**
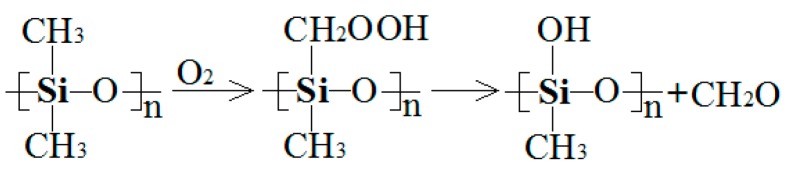
Oxidation reaction of silicone under high moisture conditions.

**Scheme 3:**
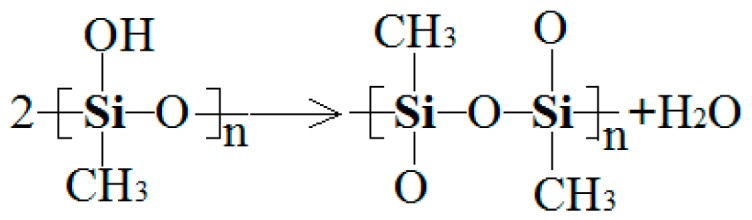
Cross-linking reaction of silicone under high moisture conditions.

Figure 16 shows the FTIR results of test samples before and after 1008 h ageing. Since FTIR can only be used to detect the polymer’s molecule structure, it is found that the FTIR spectra of pure silicone and 10% phosphor/silicone composite before ageing were basically overlapped. After ageing, some new peaks were generated in the 1687 cm^−1^ and 1457 cm^−1^ bands, corresponding to the C=O and C–H bonds, respectively. As described in Scheme 2, this may be due to the fact that O_2_ reacted with the side groups, which formed the C=O bond at the side groups due to this oxidization. However, since the side group after oxidation was not stable, a cross-linking reaction occurred, resulting in an increase in methyl CH_3_ in the molecular structure. In addition, the aged silicone produced the O-H bond as shown in 3600 cm^−1^–3500 cm^−1^, which was generated by the oxidation reaction of silicon. As summarized, the oxidation and cross-linking reaction of silicone under high moisture conditions can be expressed by Scheme 2 and Scheme 3, respectively. Meanwhile, as being detected by the SEM/EDS analysis shown in Table 3, an increase of O content in silicone after ageing also validates the oxidation phenomenon.

#### 3.2.5. SEM/EDS Result Analysis

**Figure 17:**
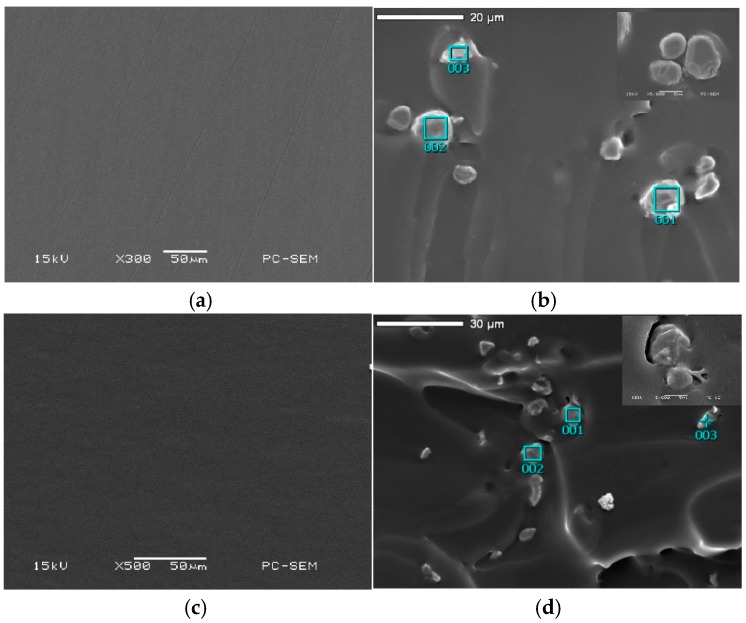
Fracture morphology of test samples: (**a**) 0% and (**b**) 10% before ageing; (**c**) 0% and (**d**) 10% after ageing.

Finally, the fracture morphology and chemical element compositions of test samples before and after 1008 h ageing are compared and shown in Figure 17. The SEM images present the fracture morphologies of samples after tensile tests, which shows some delamination happened between phosphor particles and silicone matrix after ageing. Moreover, according to the chemical element test results from EDS shown in Table 3, it is known that the main chemical composition of selected phosphor YAG04 is Y_3_Al_5_O_12_. During the ageing process, the O contents of both silicone and phosphor were increased, but their Al, Y and Si contents were reduced. This reveals that, since the moisture entered into the phosphor/silicone composite, the silicone underwent the oxidation and cross-linking reactions, and the phosphor suffered a certain degree of hydrolysis reaction which can be expressed as Equation (10) [17]. These results keep correspondence with the above FTIR results.

(10) $$Y_{3}Al_{5}O_{12}+9H_{2}O\to 5Al^{3+}+3HYO_{2}+15OH^{-}.$$

## 4. Conclusions

This paper investigates the curing mechanism of phosphor/silicone composites used in pc-WLED packages and evaluated their mechanical properties after a long-term high moisture ageing test. The simulation and experimental results of this study can be concluded as: (1) the in situ measured isothermal viscosity curves of both pristine silicone and phosphor/silicone composites follow the Arrhenius model, and high temperature and high phosphor mass fraction can increase the curing rate; (2) the hydrosilylation reaction between silicones determines the curing mechanism of phosphor/silicone composite; (3) the tensile test, FE simulation and Mori–Tanaka theoretical results conclude that there is an increase of Young’s modulus of phosphor/silicone composites when the phosphor additions increase; (4) after the long-term high moisture ageing, the Young’s modulus of composite increases more than that of pure silicone, as besides the oxidation and cross-linking reaction happening within silicone, the phosphor hydrolysis can also enhance the stiffness of composite. Thus, these research findings can not only help guide the material selection and process optimization in the pc-WLED packaging, but also effectively improve the packaging strength and reliability of pc-WLED packages.

## Figures and Tables

**Table 1:** Characteristic peaks of silicone A and B in the FTIR spectrum.

Silicone A		Silicone B	
**Vibration Frequency/cm^−1^**	**Vibration Mode**	**Vibration Frequency/cm^−1^**	**Vibration Mode**
2962, 2904	CH_3_	2962, 2904	CH_3_
1699	CH_2_=CH_2_	2167	Si–H
1415, 1259	Si–CH_3_	1415, 1259	Si–CH_3_
1100–1000	Si–O–Si	1100–1000	Si–O–Si
800	C–Si	800	C–Si

**Table 2:** FEA simulation parameters.

Components	Density (g/cm^3^)	Young’s Modulus (MPa)	Poisson’s Ratio
Silicone	1.04	3.10	0.48
Phosphor	4.56	335,000	0.28

**Table 3:** Comparison of chemical element content before and after ageing.

Components	Elements	Averaged Element Contents before Ageing (%)	Averaged Element Contents after Ageing (%)
Phosphor	O	8.91	11.32
	Al	28.18	27.57
	Y	57.37	52.01
Silicone	C	18.58	22.72
	O	5.31	8.29
	Si	76.11	69.00
